# 5-(4-Nitro­benz­yl)-1*H*-1,2,3,4-tetra­zole

**DOI:** 10.1107/S1600536810043576

**Published:** 2010-10-31

**Authors:** Jin Mei Chen, Hong Zhao

**Affiliations:** a School of Chemistry and Chemical Engineering, Southeast University, Nanjing 210096, People’s Republic of China

## Abstract

In the title compound, C_8_H_7_N_5_O_2_, the dihedral angle between the benzene and tetra­zole rings is 63.13 (8)°. The crystal structure exhibits inter­molecular N—H⋯N hydrogen bonds which lead to the formation of one-dimensional chains along the [010] direction.

## Related literature

For the applications of tetra­zoles, see: Demko & Sharpless (2001 ▶). For our previous work on this class of compounds, see: Zhao *et al.* (2008 ▶).
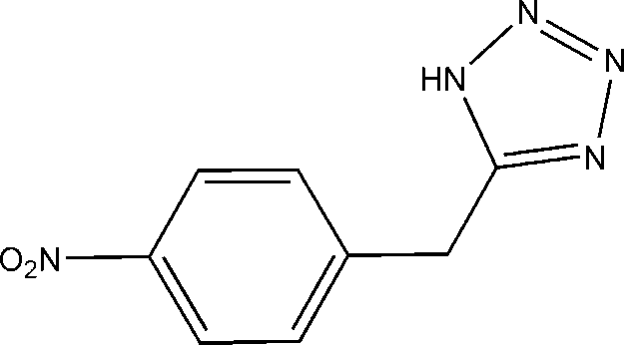


## Experimental

### 

#### Crystal data

C_8_H_7_N_5_O_2_
*M* *_r_* = 205.19
Monoclinic, 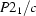

*a* = 6.3393 (10) Å
*b* = 4.9381 (6) Å
*c* = 28.801 (4) Å
β = 101.905 (14)°
*V* = 882.2 (2) Å^3^
*Z* = 4
Mo *K*α radiation
μ = 0.12 mm^−1^
*T* = 295 K
0.50 × 0.42 × 0.28 mm

#### Data collection

Rigaku SCXmini diffractometer
Absorption correction: multi-scan (*CrystalClear*; Rigaku, 2005 ▶) *T*_min_ = 0.982, *T*_max_ = 0.990
8583 measured reflections
2088 independent reflections
1823 reflections with *I* > 2σ(*I*)
*R*_int_ = 0.034

#### Refinement

*R*[*F* ^2^ > 2σ(*F* ^2^)] = 0.058
*wR*(*F* ^2^) = 0.128
*S* = 1.20
2088 reflections
138 parameters
H-atom parameters constrained
Δρ_max_ = 0.27 e Å^−3^
Δρ_min_ = −0.25 e Å^−3^

### 

Data collection: *CrystalClear* (Rigaku, 2005 ▶); cell refinement: *CrystalClear*; data reduction: *CrystalClear*; program(s) used to solve structure: *SHELXS97* (Sheldrick, 2008 ▶); program(s) used to refine structure: *SHELXL97* (Sheldrick, 2008 ▶); molecular graphics: *SHELXTL/PC* (Sheldrick, 2008 ▶); software used to prepare material for publication: *SHELXTL/PC*.

## Supplementary Material

Crystal structure: contains datablocks I, global. DOI: 10.1107/S1600536810043576/bx2321sup1.cif

Structure factors: contains datablocks I. DOI: 10.1107/S1600536810043576/bx2321Isup2.hkl

Additional supplementary materials:  crystallographic information; 3D view; checkCIF report

## Figures and Tables

**Table 1:** Hydrogen-bond geometry (Å, °)

*D*—H⋯*A*	*D*—H	H⋯*A*	*D*⋯*A*	*D*—H⋯*A*
N1—H1⋯N4^i^	0.87	1.94	2.803 (2)	176

Symmetry code: (i) 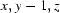.

**Fig. 1.**
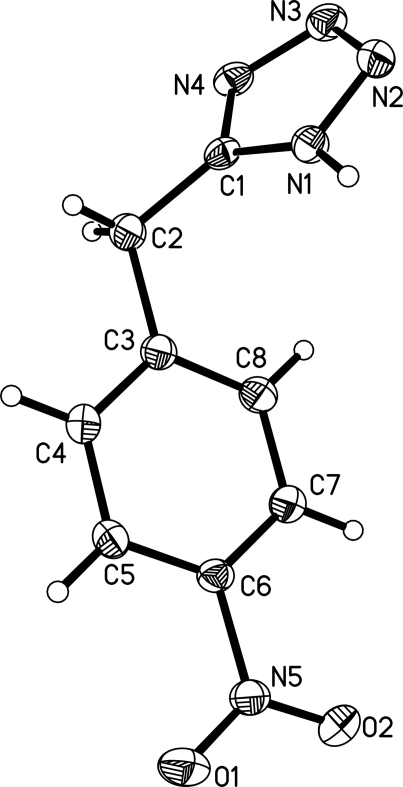
The molecular structure of the title compound, showing the atomic numbering scheme. The displacement ellipsoids are drawn at the 30% probability level.

**Fig. 2.**
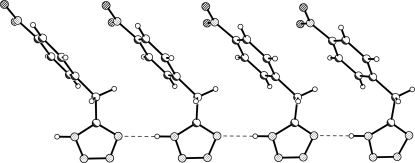
Diagram of the molecules linked into one-dimensional chain by a simple N—H···N interaction.

## supplementary crystallographic information

### Comment

Tetrazole ligands have found a wide range of application in medicine chemistry, coordination chemistry and material chemistry (Demko & Sharpless, 2001). As part of an ongoing program in our laboratory to explore the structural characterization of the tetrazole-related compounds (Zhao *et al.*, 2008), the crystal structure of the title compound is reported here.

In the molecule of the title compound (Fig. 1) bond lengths and angles have normal values. The dihedral angle between the benzene ring and tetrazole ring is 63.13 (8)°. The crystal structure exhibits intermolecular N—H···N hydrogen bonds which lead to the formation of one dimensional chains along the [010] direction. (Fig. 2; Table 1).

### Experimental

A mixture of 2-(4-nitrophenyl)acetonitrile (20 mmol), NaN_3_ (22 mmol) and NH_4_Cl (22 mmol) in DMF (15 ml) was heated at 120°C for 20 h then cooled and the solvent removed under vacuum. The residue was poured into water (20 ml) to give the crude title compound. Colourless prismatic crystals suitable for X-ray analysis were obtained by slow evaporation of a 95% ethanol/water solution.

### Refinement

All H atoms were detected in a difference map, but were placed in calculated positions and refined using a riding motion approxmation, with C—H = 0.93–0.97 Å, with *U*_iso_(H) = 1.2*U*_eq_(C); N—H = 0.87 Å, with *U*_iso_(H) = 1.5*U*_eq_(N).

### Crystal data

**Table:**

C_8_H_7_N_5_O_2_	*F*(000) = 424
*M**_r_* = 205.19	*D*_x_ = 1.545 Mg m^−^^3^
Monoclinic, *P*2_1_/*c*	Mo *K*α radiation, λ = 0.71073 Å
Hall symbol: -P 2ybc	Cell parameters from 2188 reflections
*a* = 6.3393 (10) Å	θ = 3.2–27.5°
*b* = 4.9381 (6) Å	µ = 0.12 mm^−^^1^
*c* = 28.801 (4) Å	*T* = 295 K
β = 101.905 (14)°	Prism, pale yellow
*V* = 882.2 (2) Å^3^	0.50 × 0.42 × 0.28 mm
*Z* = 4	

### Data collection

**Table:**

Rigaku SCXmini diffractometer	2088 independent reflections
Radiation source: fine-focus sealed tube	1823 reflections with *I* > 2σ(*I*)
graphite	*R*_int_ = 0.034
Detector resolution: 13.6612 pixels mm^-1^	θ_max_ = 27.9°, θ_min_ = 2.9°
CCD_Profile_fitting scans	*h* = −8→8
Absorption correction: multi-scan (*CrystalClear*; Rigaku, 2005)	*k* = −6→6
*T*_min_ = 0.982, *T*_max_ = 0.990	*l* = −37→37
8583 measured reflections	

### Refinement

**Table:**

Refinement on *F*^2^	Secondary atom site location: difference Fourier map
Least-squares matrix: full	Hydrogen site location: inferred from neighbouring sites
*R*[*F*^2^ > 2σ(*F*^2^)] = 0.058	H-atom parameters constrained
*wR*(*F*^2^) = 0.128	*w* = 1/[σ^2^(*F*_o_^2^) + (0.0293*P*)^2^ + 0.5689*P*] where *P* = (*F*_o_^2^ + 2*F*_c_^2^)/3
*S* = 1.20	(Δ/σ)_max_ < 0.001
2088 reflections	Δρ_max_ = 0.27 e Å^−^^3^
138 parameters	Δρ_min_ = −0.25 e Å^−^^3^
0 restraints	Extinction correction: *SHELXL97* (Sheldrick, 2008), Fc^*^=kFc[1+0.001xFc^2^λ^3^/sin(2θ)]^-1/4^
Primary atom site location: structure-invariant direct methods	Extinction coefficient: 0.037 (6)

### Special details

**Table:**

Geometry. All e.s.d.'s (except the e.s.d. in the dihedral angle between two l.s. planes) are estimated using the full covariance matrix. The cell e.s.d.'s are taken into account individually in the estimation of e.s.d.'s in distances, angles and torsion angles; correlations between e.s.d.'s in cell parameters are only used when they are defined by crystal symmetry. An approximate (isotropic) treatment of cell e.s.d.'s is used for estimating e.s.d.'s involving l.s. planes.
Refinement. Refinement of *F*^2^ against ALL reflections. The weighted *R*-factor *wR* and goodness of fit *S* are based on *F*^2^, conventional *R*-factors *R* are based on *F*, with *F* set to zero for negative *F*^2^. The threshold expression of *F*^2^ > σ(*F*^2^) is used only for calculating *R*-factors(gt) *etc*. and is not relevant to the choice of reflections for refinement. *R*-factors based on *F*^2^ are statistically about twice as large as those based on *F*, and *R*- factors based on ALL data will be even larger.

### Fractional atomic coordinates and isotropic or equivalent isotropic displacement parameters (Å^2^)

**Table:**

	*x*	*y*	*z*	*U*_iso_*/*U*_eq_	
O1	0.6521 (3)	−0.0440 (4)	0.23878 (6)	0.0537 (5)	
O2	0.3476 (3)	0.0031 (4)	0.18977 (6)	0.0538 (5)	
N1	0.7922 (3)	0.6457 (3)	0.02183 (6)	0.0340 (4)	
H1	0.7833	0.4706	0.0214	0.051*	
N2	0.7245 (3)	0.7302 (4)	−0.02353 (6)	0.0402 (4)	
N3	0.7205 (3)	0.9910 (4)	−0.02326 (6)	0.0413 (5)	
N4	0.7847 (3)	1.0783 (3)	0.02209 (6)	0.0358 (4)	
N5	0.5369 (3)	0.0619 (4)	0.20421 (6)	0.0367 (4)	
C1	0.8285 (3)	0.8620 (4)	0.04930 (7)	0.0297 (4)	
C2	0.9119 (4)	0.8641 (4)	0.10150 (7)	0.0399 (5)	
H2A	0.8881	1.0425	0.1135	0.048*	
H2B	1.0664	0.8337	0.1076	0.048*	
C3	0.8111 (3)	0.6555 (4)	0.12886 (7)	0.0327 (4)	
C4	0.9324 (3)	0.5398 (4)	0.16973 (7)	0.0369 (5)	
H4	1.0754	0.5918	0.1802	0.044*	
C5	0.8439 (3)	0.3485 (4)	0.19519 (7)	0.0371 (5)	
H5	0.9255	0.2715	0.2225	0.044*	
C6	0.6314 (3)	0.2747 (4)	0.17898 (7)	0.0316 (4)	
C7	0.5058 (3)	0.3865 (5)	0.13898 (7)	0.0382 (5)	
H7	0.3629	0.3337	0.1288	0.046*	
C8	0.5962 (3)	0.5790 (4)	0.11420 (7)	0.0386 (5)	
H8	0.5124	0.6585	0.0874	0.046*	

### Atomic displacement parameters (Å^2^)

**Table:**

	*U*^11^	*U*^22^	*U*^33^	*U*^12^	*U*^13^	*U*^23^
O1	0.0614 (10)	0.0516 (10)	0.0475 (9)	−0.0025 (8)	0.0098 (8)	0.0188 (8)
O2	0.0503 (9)	0.0592 (11)	0.0516 (9)	−0.0227 (8)	0.0099 (8)	0.0033 (8)
N1	0.0432 (9)	0.0243 (8)	0.0348 (9)	−0.0038 (7)	0.0085 (7)	−0.0009 (7)
N2	0.0465 (10)	0.0401 (10)	0.0332 (9)	−0.0083 (8)	0.0064 (7)	0.0007 (8)
N3	0.0388 (9)	0.0413 (10)	0.0421 (10)	−0.0031 (8)	0.0041 (8)	0.0134 (8)
N4	0.0354 (9)	0.0301 (9)	0.0430 (9)	−0.0003 (7)	0.0104 (7)	0.0087 (7)
N5	0.0476 (10)	0.0317 (9)	0.0326 (9)	−0.0050 (8)	0.0127 (7)	−0.0009 (7)
C1	0.0318 (9)	0.0234 (9)	0.0352 (10)	−0.0035 (7)	0.0102 (7)	0.0009 (7)
C2	0.0530 (12)	0.0330 (11)	0.0332 (10)	−0.0122 (9)	0.0074 (9)	−0.0023 (9)
C3	0.0406 (10)	0.0278 (10)	0.0301 (9)	−0.0033 (8)	0.0083 (8)	−0.0022 (8)
C4	0.0354 (10)	0.0395 (11)	0.0336 (10)	−0.0073 (9)	0.0021 (8)	−0.0012 (9)
C5	0.0408 (11)	0.0385 (11)	0.0290 (9)	−0.0031 (9)	0.0006 (8)	0.0024 (8)
C6	0.0400 (10)	0.0268 (9)	0.0292 (9)	−0.0033 (8)	0.0102 (8)	−0.0006 (7)
C7	0.0333 (10)	0.0439 (12)	0.0365 (10)	−0.0045 (9)	0.0048 (8)	0.0031 (9)
C8	0.0389 (10)	0.0395 (12)	0.0350 (10)	−0.0010 (9)	0.0018 (8)	0.0090 (9)

### Geometric parameters (Å, °)

**Table:**

O1—N5	1.224 (2)	C2—H2B	0.9700
O2—N5	1.221 (2)	C3—C4	1.389 (3)
N1—C1	1.321 (2)	C3—C8	1.392 (3)
N1—N2	1.354 (2)	C4—C5	1.384 (3)
N1—H1	0.8666	C4—H4	0.9300
N2—N3	1.288 (3)	C5—C6	1.381 (3)
N3—N4	1.356 (3)	C5—H5	0.9300
N4—C1	1.320 (2)	C6—C7	1.374 (3)
N5—C6	1.473 (2)	C7—C8	1.382 (3)
C1—C2	1.487 (3)	C7—H7	0.9300
C2—C3	1.516 (3)	C8—H8	0.9300
C2—H2A	0.9700		
			
C1—N1—N2	108.07 (16)	C4—C3—C8	118.78 (18)
C1—N1—H1	145.0	C4—C3—C2	120.03 (18)
N2—N1—H1	106.6	C8—C3—C2	121.18 (18)
N3—N2—N1	107.76 (17)	C5—C4—C3	121.02 (18)
N2—N3—N4	108.73 (16)	C5—C4—H4	119.5
C1—N4—N3	107.42 (16)	C3—C4—H4	119.5
O2—N5—O1	123.73 (18)	C6—C5—C4	118.38 (18)
O2—N5—C6	118.18 (17)	C6—C5—H5	120.8
O1—N5—C6	118.08 (17)	C4—C5—H5	120.8
N4—C1—N1	108.02 (17)	C7—C6—C5	122.25 (18)
N4—C1—C2	125.59 (18)	C7—C6—N5	118.43 (17)
N1—C1—C2	126.35 (17)	C5—C6—N5	119.28 (17)
C1—C2—C3	114.87 (16)	C6—C7—C8	118.63 (19)
C1—C2—H2A	108.6	C6—C7—H7	120.7
C3—C2—H2A	108.5	C8—C7—H7	120.7
C1—C2—H2B	108.6	C7—C8—C3	120.92 (18)
C3—C2—H2B	108.6	C7—C8—H8	119.5
H2A—C2—H2B	107.5	C3—C8—H8	119.5
			
C1—N1—N2—N3	0.1 (2)	C3—C4—C5—C6	0.0 (3)
N1—N2—N3—N4	−0.2 (2)	C4—C5—C6—C7	−0.7 (3)
N2—N3—N4—C1	0.2 (2)	C4—C5—C6—N5	177.08 (18)
N3—N4—C1—N1	−0.2 (2)	O2—N5—C6—C7	−3.4 (3)
N3—N4—C1—C2	177.72 (18)	O1—N5—C6—C7	177.28 (19)
N2—N1—C1—N4	0.1 (2)	O2—N5—C6—C5	178.74 (19)
N2—N1—C1—C2	−177.80 (18)	O1—N5—C6—C5	−0.5 (3)
N4—C1—C2—C3	138.7 (2)	C5—C6—C7—C8	0.1 (3)
N1—C1—C2—C3	−43.8 (3)	N5—C6—C7—C8	−177.64 (19)
C1—C2—C3—C4	147.51 (19)	C6—C7—C8—C3	1.1 (3)
C1—C2—C3—C8	−33.4 (3)	C4—C3—C8—C7	−1.7 (3)
C8—C3—C4—C5	1.1 (3)	C2—C3—C8—C7	179.3 (2)
C2—C3—C4—C5	−179.8 (2)		

### Hydrogen-bond geometry (Å, °)

**Table:**

*D*—H···*A*	*D*—H	H···*A*	*D*···*A*	*D*—H···*A*
N1—H1···N4^i^	0.87	1.94	2.803 (2)	176

Symmetry codes: (i) *x*, *y*−1, *z*.
